# Matricial support for community health agents on the auditory and language development milestones in early childhood

**DOI:** 10.1590/2317-1782/20232021135en

**Published:** 2023-09-01

**Authors:** Lidiane Assis da Silva, Vanessa de Lima Silva

**Affiliations:** 1 Programa de Residência Multiprofissional em Saúde da Família, Centro de Ciências da Saúde, Universidade Federal de Pernambuco - UFPE - Recife (PE), Brasil.; 2 Departamento de Fonoaudiologia, Centro de Ciências da Saúde, Universidade Federal de Pernambuco - UFPE - Recife (PE), Brasil.

**Keywords:** Speech, Primary Health Care, Community Health Workers, House Calls, Child Health

## Abstract

**Purpose:**

to analyze the knowledge of community health agents about hearing and language development milestones in early childhood, in the context of home visits, before and after a team cooperation workshop.

**Methods:**

action research was developed, with a quantitative and qualitative approach.

**Results:**

Significant contribution to the agents’ learning, enriching their home visit practices.

**Conclusion:**

Team cooperation is an important tool to qualify the performance of community health agents regarding guidelines on hearing and language milestones in early childhood.

## INTRODUCTION

The main work instrument in speech-language-hearing (SLH) therapy is human communication, which is essential to people’s full development. Because of the importance of communication to their thorough development, it is essential to have SLH therapists in primary healthcare (PHC), as they are the main rehabilitation professionals providing such care. Communication disorders interfere directly with various contexts in life, including physical, sensory, psychological, and social ones^(1)^.

SLH therapists’ work in PHC is a recent construction full of challenges. It is mainly based on the principles of the Unified Health System (SUS), aiming to provide the whole population with greater care in the areas of voice, language, hearing, and oral-motor control, improving their quality of life^(2)^.

SLH therapists are responsible along with the multidisciplinary PHC team for various activities, such as situational and institutional diagnoses, support, home visits, individual and/or group treatment, health campaigns, permanent education for the teams, and conducting and publishing research^(3)^.

Language is one of the areas addressed by SLH therapy, with an essential role in perceptual organization, information reception and structuring, and social interaction. Moreover, hearing is a prerequisite to acquiring and developing language, so hearing loss is one of the main disorders that interfere with language and speech development, as hearing and language are correlated and interdependent functions^(4)^.

In 2008, the Ministry of Health Regulation no. 154 created the Extended Center for Family Health and Basic Care (NASF-AB, in Portuguese) to meet these needs and provide comprehensive healthcare, aiming to broaden PHC coverage and scope of activity. Their teams have professionals from different fields of knowledge, who are expected to work in cooperation, support the Family Health Teams, and share practices and knowledge about health in the regions under their responsibility^(5)^.

The work of NASF-AB must be guided by the theoretical-methodological framework of team cooperation. Such support is an innovative means of producing health, incorporating interdisciplinary practices, and ensuring comprehensive attention throughout the health system^(6)^.

Home visits are unique care opportunities, aiming to promote health in the community in a setting other than the health unit. It uses light technology, ensuring more humanized and supportive care, and building rapport between professionals, users, the family, and the community^(7)^. SLH therapy provided through home visits is an important tool to promote the population’s health.

The Ministry of Health uses development milestones as references to follow up on children in neonatal consultations - whose periodicity in health units, involving weight and length control, justifies including hearing and language development follow-up. Another possibility is to apply such instruments in home visits^(8)^.

The Ministry of Health has indicated the following milestones involving hearing and language behaviors: reacting to sounds, emitting sounds, locating sounds, emitting sounds, emitting sounds, laughing aloud, duplicating syllables, producing “jargons”, speaking one word, speaking three words, and speaking 2-word sentences^(9)^.

Community health agents (CHA) are the target public of NASF-AB team cooperation. They can provide base support to children’s hearing loss and language changes prevention, diagnosis, and intervention. These professionals are the link between SUS and the community, identifying risks or problems that might interfere with hearing and language development, thus referring them when necessary^(10)^.

CHA training provides a broader knowledge of SLH therapy and team cooperation through home visits regarding hearing and language development in early childhood, reaching the scientific community, health professionals, administrators, and the population that uses SLH therapy at SUS.

Given the above, this article aimed to analyze CHA’s knowledge of hearing and language development milestones in early childhood, in the context of home visits, before and after team cooperation workshops. The perspective is to contribute to scientific advances on the said issue, considering the few such studies in the scientific literature.

## METHOD

This study was approved by the Research Ethics Committee of the Department of Health Sciences at the Federal University of Pernambuco, under evaluation report no. 4.147.895.

Qualitative and quantitative approaches were used in this action survey, which is a type of empirical social survey conceived and carried out in close association with an action or the solution to a public problem. In it, researchers and participants who represent the situation or problem are mutually cooperative or participative^(11)^.

This study approached the region covered by the Family Health Unit of Vila União, in Health District IV of the municipality of Recife, Pernambuco, Brazil. The unit has four Family Health Teams.

Eighteen CHAs participated in the quantitative stage of the research, while four CHAs participated in its second (qualitative) stage, with the volunteer participation of one CHA from each team. The second stage only had four participants because it was conducted during the pandemic. They were recruited by invitation to participate in the research.

Participants were informed of the study objectives and data collection procedures both verbally and in writing and had their questions answered. Those who agreed to participate signed an informed consent form.

The inclusion criteria were as follows: having worked for more than 1 year as CHA in the Family Health Unit approached in the study, being interested in and available to participate in the research. CHAs who were on a leave of absence or vacation during data collection were excluded.

Data were collected in three phases: Situational diagnosis; Action; and Action assessment. Initially, the situational diagnosis was conducted through a structured interview form to gather data on the research participants’ knowledge, attitudes, and practices (KAP) regarding hearing and language development milestones in early childhood.

The data collection instrument was developed by the researchers, using references for each dimension in the KAP method, as follows:

Knowledge refers to recalling specific facts (in the education system to which the person belongs) or skills to solve specific problems or define concepts based on the understanding they have acquired on a given topic or event. Attitude means having relatively unceasing opinions, willingness, beliefs, feelings, and affectivity about things, situations, or people. It is related to the affective dominion and portrays the emotional dimension. Practice is the decision to do an action. It is related to the cognitive and psychomotor domains and reinforces the social dimension^(12:25)^.

The form had two sections. The first one comprised sociodemographic and professional variables, and the second one had the KAP variables.

A focus group with participating CHAs was also conducted before and after the workshop, at the Family Health Unit in Vila União.

Focus groups are interviews with few participants on a specific topic, emphasizing the attention to various opinions. Each person is encouraged to share their ideas and listen and respond to those of the other ones, aiming at people’s synergy, rather than consensus. Focus-group research is also centered on discussion, more in-depth information thanks to group interaction and influence, low cost, easy-to-understand methods and results, noticeable differences and contradictions in opinions between participants, and quick results^(13)^.

The group was facilitated by a moderator, while another person took notes of the statements. The group’s responses were recorded and later transcribed.

Participants were asked guiding questions, organized into four analysis themes. If the group did not respond satisfactorily, auxiliary questions were asked.

Interviewees were identified as CHA and the interview number to ensure their anonymity. For instance, the first CHA to be interviewed was identified as CHA 1, the second one as CHA 2, and so forth.

The action in this research was developed in a team cooperation workshop lasting about 4 hours, encouraging CHAs’ autonomy to construct knowledge. It included a lecture and discussions about hearing and language development milestones and the CHAs’ procedures in home visits after identifying these milestones. The program included team-building activities to both relax and involve participants in the pedagogical approach, encouraging their participation and reflection on the central workshop theme (Chart 1).

**Chart 1:** Team cooperation workshop program, approaching hearing and language development milestones in early childhood with community health agents

**Topic: “Hearing and language development milestones”**
Learning goals:
Address hearing and language development milestones.
Address home visit procedures after identifying development milestones.
Material:
a) Card stock.
b) Labels.
c) Pens.
**1^st^ moment: Sharing knowledge about hearing and language development milestones in early childhood**
A group activity was initially carried out to, pasting labels on card stock. The CHAs were shown various paper labels with the hearing and language milestones. Then, they were asked to stick in the right place the ones related to hearing and the ones related to speech development.
**2^nd^ moment: Discussing hearing and language development milestones in early childhood**
The discussion was based on the CHAs’ previous knowledge. They sat in a circle, using the card stock of the 1^st^ moment to begin talking about each development milestone.
**3^rd^ moment: Sharing about home visit procedures after identifying development milestones**
This moment was based on examples of problem situations CHAs had experienced on their everyday home visits. According to the reports, they discussed the necessary procedure.
**4^th^ moment: Closing group activity**
Each participant defined the workshop in one word.

Data collection finished with a new focus group after the workshop, carried out at the Family Health Unit in Vila União. It followed the same model as the first one, using the same guiding questions, and adding other ones on the process experienced in the workshop.

Qualitative data were submitted to content analysis, as proposed by Bardin^(14)^. It had the following stages: skimming the transcriptions to understand general content and organize data; identifying categories and subcategories to analyze the subjects’ statements; describing results; and interpreting results (addressing the relationship between analysis categories and subcategories and the study objectives). The KAP questionnaire was analyzed with descriptive statistics (distribution of absolute and relative frequencies). All data were tabulated and calculated in 2007 Microsoft Excel.

## RESULTS

Altogether, 18 CHAs participated in the research, all of them females. The predominating age range was from 41 to 50 years (88.9%). Most of them had finished high school (72.2%), and about 88.9% had been working in this position for 9 or more years (Table 1).

**Table 1:** Sociodemographic and professional characteristics of community health agents (n = 18). Recife, Pernambuco, 2020

**Variables**	**N**	**%**
**Sex**		
Males	0	0
Females	18	100%
**Age range (in years)**		
Up to 40 years	0	0
41 to 50 years	16	88.9%
More than 60 years	2	11.1%
**Educational attainment**		
Finished middle school	1	5.6%
Unfinished high school	1	5.6%
Finished high school	13	72.2%
Unfinished higher education	1	5.6%
Finished higher education	2	11.1%
**Time working as CHA (in years)**		
6 to 8 years	2	11.1%
9 or more years	16	88.9%
**TOTAL**	**18**	**100%**

The assessment of the CHAs’ KAP regarding hearing and language development milestones in early childhood showed their knowledge of the topic was fragile, and their practices were not based on evidence (Table 2).

**Table 2:** Knowledge, attitudes, and practices of community health agents regarding hearing and language development milestones in early childhood. Recife, Pernambuco, 2020

**VARIABLES**	**KAP RESPONSE**			
	**AFFIRMATIVE**		**NEGATIVE**	
	**n**	**%**	**n**	**%**
**KNOWLEDGE**				
Infant hearing screening test	17	94.12%	1	5.88%
Infant tongue screening test	9	50%	9	50%
Hospitals perform infant hearing screening tests	18	100%	0	0%
Hospitals perform infant tongue screening test	14	71.43%	4	28.57
CHAs’ responsibilities include public health actions regarding hearing and language in their region and in the waiting room.	15	83.3%	3	16.7%
They know that children’s speech development may be related to their hearing health.	18	100%	0	0%
They know that children’s speech development may be related to the stimulation they receive.	18	100%	0	0%
**ATTITUDES**				
**Sought training regarding hearing and language development milestones in early childhood**	8	44.4%	10	55.6%
**Among those who sought it, where they acquired the information**				
Speech-language-hearing therapists in the NASF-AB team	10	55.6%	8	44.4%
Pamphlets, television, books, Internet	8	44.4%	10	55.6%
**Feels trained to instruct about hearing and language development milestones in early childhood**	6	33.3%	12	66.7%
**Is interested in learning about hearing and language development milestones in early childhood**	18	100%	0	0%
**PRACTICES**				
Conducts actions to promote hearing health and language development in early childhood.	8	44.4%	10	55.6%
Uses hearing and language development milestones indicated in the personal child health record.	4	22.2%	14	77.8%
Instructs about risk factors for hearing health in early childhood.	13	72.2%	5	27.8%
Instructs about risk factors for language development in early childhood.	15	83.3%	3	16.7%
Verifies whether parents have any complaints regarding their children’s hearing or language during home visits.	16	88.9%	2	11.1%
Identifies when users need speech-language-hearing assistance.	18	100%	0	0%
When they suspect a child has communication difficulties, they discuss the signs they noticed with their team to refer them to the speech-language-hearing therapist at NASF-AB	18	100%	0	0%
Feels the need to learn about hearing and language development milestones.	18	100%	0	0%

Four of the participating CHAs also took part in the second (qualitative) phase of the research. Statement content analysis about early childhood before the team cooperation workshop identified fragile knowledge of the topic (Chart 2). They related early childhood to the children’s ages, referring only to the chronological aspect of this stage, and citing different age ranges. They did not go beyond this concept to mention characteristic elements of this phase.

**Chart 2:** Analysis categories before and after the team cooperation workshop, according to core ideas regarding the agents’ knowledge of hearing and language development milestones in early childhood

**Core ideas**	**Analysis categories before team cooperation**	**Analysis categories after team cooperation**
Knowledge about early childhood	Children’s age	Following up on children from 0 to 5 years old.
	0 to 5 years old	Following up on children from pregnancy to 5 years old.
	0 to 10 years old	
Actions aimed at early childhood in home visits	Instructions to families	Instruction to families
		Observing speech and hearing
		Listening to the parents, observing, instructing, and referring.
Instruction for the families of children in early childhood	Vaccination card	Vaccination card
	Neonatal care	Neonatal care
	Breastfeeding	Breastfeeding
	Diet	Diet
	Development milestones (crawling and babbling)	Development milestones indicated in the vaccination card
Knowledge of hearing development milestones	Nonexistent	Identifying sounds and locating sounds
Questions on hearing development in home visits	No questions were asked in home visits.	Asking the mother whether the child reacts when they hear a louder noise
	Do loud sounds or noise startle them?	
	Questions focused on the child’s speech	
Families’ questions about hearing development	No questions asked by the families.	Not applicable
	They report more questions related to language (speech)	
Knowledge of language development milestones	Nonexistent	The child says 1 or 2-syllable words.
		The child says 2-word sentences ‘’mama water’’.
	Tongue-tie	Repeats 2-syllable words (mama, papa)
		Emits sounds, emits sounds and laughs aloud
Questions on language development in home visits	No questions were asked in home visits.	Is your child emitting sounds? What type of sounds? Can they say mama, papa? Do they say gaga?
	Questions asked according to the child’s age.	If they already speak, what do they say?
	Development milestone (babbling, saying mama, papa)	Have you examined your child to check if they can hear and speak well?
		I ask: “Do they emit any sounds? Any noise? Do they call mommy or daddy?
Families’ questions about language development	Some families ask questions, others do not.	Not applicable
	When parents perceive identify something, they ask questions	
CHAs procedures when children do not reach development milestones	Referring the case to the nurse or physician.	Instruct the family and present the case to the team.
	Referring cases in NASF-AB + Family Health Team meetings	Present the case to the health team and then refer to NASF-AB
	Scheduling appointments with the physician	Present the case to my nurse or physician.
		Instruct, refer and monitor
Did this team cooperation workshop contribute to your professional practice?	Not applicable	Further knowledge.
		More knowledge.
		It helped me learn.
		Confidence
		It enriched my knowledge

After participating in the workshop, they continued to associate age with early childhood, but they gave more assertive statements on following up on children from 0 to 5 years old, as early as pregnancy.

They identified the vaccination cards as a main reference, although they had fragmented knowledge of what they received mainly in the context of health.

They also reported instructing families in the region they covered as part of actions aimed at them and early childhood.

After participating in the workshop, statements about instructions were accompanied by their observation of speech and hearing, leading to referrals.

Listening to the parents, observing, instructing, and providing referrals, which in our case is to schedule a visit to professionals in the health unit *(CHA 2).*

Concerning the type of instruction they gave, they reported the vaccination card, neonatal care, and diet. After the workshop, the statements had similar content, adding statements on development milestones available in the personal child health record for CHAs to instruct the families.

BEFORE:

Well... first, we have to instruct them about breastfeeding, right? The importance of breastfeeding and all; we instruct them to go to the unit regularly when they have a neonatal care appointment to follow up on the child’s development and instruct about baby bottles, pacifiers, and such. We also check the vaccination card to see if it’s all up to date *(CHA 1).*

AFTER:

We have to instruct them, right? Always instruct the parents to check the vaccination card often and the child’s development and growth, right? We have to instruct them and refer the children if we identify any… what is it called? Some sign, right? In the child, in their speech or hearing *(CHA 1).*

The CHAs’ responses did not refer to their knowledge about hearing development milestones. However, after participating in the workshop, they speak about identifying sounds and locating sounds as hearing milestones.

BEFORE:

No, I don’t remember *(CHA 3).*

AFTER:

Two of them are locating sounds and identifying sounds *(CHA 3).*

Their statements also lack questions on hearing during home visits, as CHAs reported asking more about language. The families likewise did not ask about hearing. The questions are always related to language.

After participating in the workshop, CHAs reported asking during home visits the mother’s perception of the child’s reactions and responses when they heard louder sounds or whether any sound startled them.

BEFORE:

Usually, I ask, we ask more about speech, right? Sometimes we ask if any noise or loud sound startles the child, but we usually ask more if the child is speaking or walking *(CHA 1).*

AFTER:

We ask the mother how the child’s doing. We ask the parents if the child gives any sign that they’re hearing when they clap their hands, if the child reacts when something falls on the floor, how’s their expression when someone’s talking, their eyes, and how they move their head. We ask parents to draw some conclusions *(CHA 2).*

The CHAs also reported not knowing about language development milestones. Once again, the statements revealed flaws in their knowledge of the topic. After participating in the workshop, they spoke about some language milestones, showing that they had then acquired such knowledge.

BEFORE:

Saying papa, pepe, dada, want, you know? We also pay attention to see if they’ll have any progress or continue like that, or even only point at things *(CHA 4).*

AFTER:

They’re eight milestones, like… what are they, again? Emitting sounds, emitting sounds and laughing aloud, speaking one word, speaking duplicated words; by five years old, they already speak sentences, complete sentences we can understand *(CHA 3).*

They said they did not always ask about language during home visits, and when they did, the questions were asked according to the child’s age. After participating in the workshop, they referred to questions on whether the child emitted sound and spoke words.

BEFORE:

To be honest, not me. No, I never asked that *(CHA 1).*

AFTER:

The mother or another adult responsible for the child, right? I’ll come up with a name! Mary, is your child emitting sounds? What type of sounds? Can they say mama, papa? *(CHA 2).*

They reported that many parents ask questions, while others do not, and that questions come up mostly when they identify something in their children.

In my area, it depends; some families ask questions, right? ‘Ana, see, he’s such and such years old, he’s already learning dirty language’. Other families do not even so; they think it’s normal not to speak. Not all of them ask, just some do. And I think the ones that ask know a little bit more, they are the ones who want more information *(CHA 3).*

Concerning the CHAs’ procedure when they found a child who had not reached development milestones, they reported referring them to the team’s nurse or physician, and that they scheduled appointments for the physician to assess that child and then refer them to the NASF-AB team. Moreover, the CHAs do not have any educational project on the topic in the unit.

After the workshop, the CHAs reported first instructing families and then referring cases to their team’s nurse or physician and then to the NASF-AB team.

BEFORE:

We refer them to the nurse or physician, and they get in touch with NASF *(CHA 2).*

AFTER:

I present the case to my physician or nurse, right? Then, when they meet with the NASF team, we mention the case again and follow up on the process, you know? We always go to their house to check the child’s development and growth, right? *(CHA 1).*

Assessments before and after the workshop revealed differences in the CHAs’ knowledge of hearing and language development milestones. All CHAs stated that the experience contributed to their learning, further enriching home visit practices with the population in their first years of life. An aspect that stood out was the lack of difference in analysis categories on hearing and language development before and after the workshop.

## DISCUSSION

All CHAs participating in the study were females, above 41 years old, having finished high school, and working in the position for more than 9 years. The predominance of females was verified in similar study results, in which females were prevalent, in the age range above 40 years^(15,16)^.

Concerning the CHAs’ KAP about hearing and language development milestones in early childhood, there were some flaws in their knowledge, and their practices were not based on evidence. CHAs must have conceptual and attitudinal skills regarding human communication health.

It has been observed that CHAs have great knowledge, but they also need training regarding SLH issues^(17)^. Thus, they need to be equipped to increase their potential of providing education, promotion, and prevention in human communication.

The findings reveal that the team cooperation process with CHAs is essential to ensure that they have more qualified and engaged practice with service users and their needs. PHC must encourage their teams to share knowledge, prevent and promote health, and improve professionally to ensure comprehensive care to the population.

Corroborating the perspective that team cooperation is essential to CHAs, a study stated that team cooperation leads to changes in the professionals’ understanding and practice, as well as organizational changes in the Family Health Units and their relationship with the service network, thus demonstrating that team cooperation is an effective intervention tool^(18)^.

Before participating in the team cooperation workshop, the CHAs’ knowledge of early childhood had a fragilized perspective of concepts acquired mainly in the context of health. It must be highlighted that early childhood does not refer to a date, but a comprehensive and specific development process people undergo at a certain time of their lives.

Corroborating this aspect, the National Policy for Comprehensive Child Healthcare stated that early childhood is the part of life from 0 to 5 years old, or until turning 6 years old. Hence, to ensure that this stage is healthy, their development must be followed up with actions on all attention levels - promotion, protection, appointments, early detection, and rehabilitation of changes that may have consequences in their future lives^(19)^. Therefore, CHAs’ everyday practice must reflect their knowledge of the said Policy, as it guides child healthcare practices.

These aspects were verified in the CHAs’ statements. These answers varied when asked about hearing, language, and following up on children and their families in home visits. Specifically, they seldom asked about hearing and more often asked about speech issues.

Scientific contributions have referred to CHAs’ reports on their approach in home visits regarding speech development, learning difficulties, and attention and concentration difficulties^(20)^. The study also points out that CHAs are responsible for following up on children’s health in home visits, based on the personal child health record, which makes it possible to verify in detail their hearing and language development.

Hence, it is necessary to rethink the priority and importance teams give to this topic, how and how often it is brought up in discussions, the team cooperation methods involving CHAs, and the responsibilities they must carry out based on such team dialogue. CHAs are a unique category, as they are inserted in and belong to the community, having direct contact with the families. In this sense, their core information was expected to be more consistent.

On the other hand, it is important to avoid blaming these professionals, as much is demanded from them. Corroborating this perspective, a study presented CHAs’ reported overwork as a difficulty to their home visits, which may help justify their difficulty carrying out their duties effectively in the aspects analyzed in this study^(21)^. Moreover, there is a reported lack of clarity on the part of the teams regarding CHAs’ main responsibilities, reallocating them to other activities that require their time and hinder them from making home visits, as recommended, not to mention that some cases require even more visits.

The responsibilities given to CHAs must also be reviewed to better define their role and dimension their actions, according to available resources, particularly avoiding deviating them from their functions^(22)^. Despite the fragilities - overwork, deviated functions, and lack of training -, CHAs provide guidance as they have contact with children and notice the possibility of interventions. However, there is scarce prevention based on previously informing the children’s mothers.

It was also identified how CHAs refer cases to their health team and then to the NASF-AB team. This shows a fragility in the work organization between CHAs and Family Health Teams, as team discussions about cases were not reported. A study indicates that the work organization of Family Health Teams must be reviewed to provide CHAs with more room for dialogue with other team members^(22)^. Thus, horizontalized teamwork integrating its various members reflects positively on the CHAs’ work.

One of the limitations of this study was the difference in the number of CHAs participating in the KAP questionnaire and focus groups. Moreover, there is little scientific literature on the topic, addressing the knowledge of CHAs in the team cooperation process to identify hearing and language development milestones in early childhood, in the context of home visits.

It is essential to conduct further research on this topic to follow up on hearing and language development in early childhood. Thus, new studies should be conducted to equip increasingly more CHAs regarding development milestones.

## CONCLUSION

The results of this study allow for inferences on the CHAs’ fragile knowledge about hearing and language development milestones in early childhood. Such knowledge, however, increased after the workshop. One aspect to highlight is that CHAs did not feel trained to instruct about hearing and language milestones. On the other hand, it verified the need to update professionals on the topic, thus stating the importance of team cooperation to these professionals.

It is necessary to debate the CHAs’ knowledge to identify hearing and language development milestones in early childhood, as they have privileged contact with the population and can perceive possible hindrances to children’s hearing and speech development in their earliest years.
