# Supplementary material for: Matricial support for community health agents on the auditory and language development milestones in early childhood
Source: Codas. 2023 Sep 1;35(6):e20210135. doi: 10.1590/2317-1782/20232021135en (PMC10547136; doi:10.1590/2317-1782/20232021135en)
Supplement: Translation

# Translation: Apoio matricial para agentes comunitários de saúde sobre os marcos do desenvolvimento auditivo e de linguagem na primeira infância

**DOI:** 10.1590/2317-1782/20232021135pt

**Authors:** Lidiane Assis da Silva, Vanessa de Lima Silva

**Affiliations:** 1 Programa de Residência Multiprofissional em Saúde da Família, Centro de Ciências da Saúde, Universidade Federal de Pernambuco - UFPE - Recife (PE), Brasil.; 2 Departamento de Fonoaudiologia, Centro de Ciências da Saúde, Universidade Federal de Pernambuco - UFPE - Recife (PE), Brasil.

**Keywords:** Fonoaudiologia, Atenção Primária à Saúde, Agentes Comunitários de Saúde, Visita Domiciliar, Saúde da Criança

## Abstract

**Objetivo:**

analisar o conhecimento dos agentes comunitários de saúde (ACS) sobre os marcos do desenvolvimento auditivo e de linguagem na primeira infância, no contexto da visita domiciliar, antes e após oficina de apoio matricial.

**Método:**

Foi desenvolvida uma pesquisa-ação, com abordagem quantitativa e qualitativa.

**Resultados:**

Contribuição significativa para a aprendizagem das ACS enriquecendo a prática da visita domiciliar.

**Conclusão:**

O apoio matricial é uma importante ferramenta para a atuação qualificada das ACS no tocante às orientações sobre os marcos auditivos e de linguagem na primeira infância.

## INTRODUÇÃO

A Fonoaudiologia tem como seu principal instrumento de trabalho a comunicação humana. Esta por sua vez é fundamental para o pleno desenvolvimento das pessoas. Considerando a importância da comunicação para o desenvolvimento integral do indivíduo, o fonoaudiólogo é um profissional imprescindível na Atenção Básica à saúde, sendo ele o principal profissional reabilitador para prestar este cuidado. Na existência de distúrbios na comunicação, há interferência diretamente em diversos âmbitos da vida das pessoas, sejam eles físicos, sensoriais, psicológicos e sociais^(1)^.

Sendo assim a atuação do fonoaudiólogo na Atenção Primária à Saúde (APS) é uma construção recente e cheia de desafios. Essa atuação baseia-se primeiramente nos princípios do Sistema Único de Saúde (SUS), que visa oferecer a toda população um cuidado maior com as áreas da voz, linguagem, audição e motricidade orofacial, favorecendo uma melhor qualidade de vida^(2)^.

Na APS cabe ao fonoaudiólogo junto à equipe multidisciplinar, realizar diversas atividades, algumas são: diagnóstico situacional e institucional; acolhimento; visitas domiciliares; atendimentos individualmente e/ou em grupos; atuação em campanhas de saúde; educação permanente das equipes, bem como a realização e divulgação de pesquisas^(3)^.

A linguagem é uma das áreas de atuação da fonoaudiologia, no qual o seu papel é fundamental na organização perceptual, na recepção e estruturação das informações e interações sociais. Além disso, temos a audição constituindo-se como um pré-requisito para a aquisição e desenvolvimento da linguagem. Tendo como um dos principais distúrbios que pode interferir no desenvolvimento da linguagem e da fala, a deficiência auditiva. Onde a audição e a linguagem são funções correlacionadas e interdependentes^(4)^.

Com o intuito de solucionar essas demandas e alcançar a atenção integral à saúde, o Núcleo Ampliado de Saúde da Família e Atenção Básica (Nasf-AB), foi criado em 2008, pela portaria 154 do Ministério da Saúde (MS), objetivando ampliar a abrangência e o escopo das ações de atenção básica. Suas equipes são compostas por profissionais de diferentes áreas de conhecimento, que devem atuar de forma integrada, apoiando as Equipes de Saúde da Família (EqSF), compartilhando as práticas e saberes em saúde nos territórios sob responsabilidade destas equipes^(5)^.

O Nasf-AB deve ter seu trabalho orientado pelo referencial teórico-metodológico do apoio matricial. Uma vez que este apoio proporciona a produção de saúde de maneira inovadora com a incorporação da prática interdisciplinar, garantindo a integralidade da atenção em todo o sistema de saúde^(6)^.

A visita domiciliar é uma oportunidade diferente de cuidado que visa promover saúde à comunidade, desenvolve-se em um espaço externo à unidade de saúde. Caracteriza-se por fazer uso de uma tecnologia leve, permitindo o cuidado de forma mais humana, acolhedora, estabelecendo laços de confiança entre profissionais e usuários, a família e a comunidade^(7)^. A atuação da fonoaudiologia através de visitas domiciliares apresenta-se como uma importante ferramenta de promoção à saúde da população.

O MS utiliza marcos do desenvolvimento como referencial para o acompanhamento das crianças durante as consultas de puericultura. Considerando a periodicidade deste acompanhamento realizado na unidade de saúde, no qual envolve o controle de peso e a estatura, o acompanhamento do desenvolvimento auditivo e de linguagem podem ser incluídos nestas consultas, ou ainda, durante as visitas domiciliares pode aplicar estes instrumentos^(8)^.

Os marcos sugeridos pelo MS que envolvem os comportamentos auditivos e da linguagem são: reage ao som, emite sons, localiza o som, emite sons, emite sons, ri alto, duplica sílabas, produz “jargão”, fala uma palavra, fala 3 palavras, fala frases com 2 palavras^(9)^.

Os Agentes Comunitários de Saúde (ACS) são o público-alvo para as ações de apoio matricial do Nasf-AB. Eles podem fornecer um apoio basilar à prevenção, diagnóstico e intervenção na deficiência auditiva e nas alterações de linguagem da criança. Estes profissionais são o vínculo entre o SUS e a comunidade. Atuando como os identificadores e encaminhadores (quando necessário) de riscos ou problemas que possam interferir no desenvolvimento auditivo e de linguagem^(10)^.

A formação dos ACS promove um conhecimento mais amplo acerca da atuação da fonoaudiologia no apoio matricial sobre o desenvolvimento auditivo e de linguagem na primeira infância, no contexto da visita domiciliar, tanto para a comunidade científica, para os profissionais dos serviços de saúde, gestores e população usuária da Fonoaudiologia no SUS.

Diante do exposto, este artigo objetivou analisar o conhecimento dos ACS sobre os marcos do desenvolvimento auditivo e de linguagem na primeira infância, no contexto da visita domiciliar, antes e após a oficina de apoio matricial. A perspectiva é colaborar com o avanço científico sobre a problemática acima exposta, tendo em vista a escassez de estudos na literatura científica.

## MÉTODO

O presente estudo foi aprovado pelo Comitê de Ética em Pesquisas do Centro de Ciências da Saúde da Universidade Federal de Pernambuco, sob o número do Parecer: 4.147.895.

Foram conduzidas abordagens metodológicas qualitativa e quantitativa, com desenho do tipo pesquisa-ação. Sendo a pesquisa-ação um tipo de pesquisa social com base empírica que é concebida e realizada em estreita associação com uma ação ou a resolução de um problema coletivo e no qual os pesquisadores e os participantes representativos da situação ou do problema estão envolvidos de modo cooperativo ou participativo^(11)^.

A área do presente estudo refere-se ao território coberto pela Unidade de Saúde da Família de Vila União, localizada no Distrito Sanitário IV do município do Recife - PE. A unidade possui quatro Equipes de Saúde da Família.

Participaram da etapa quantitativa da pesquisa 18 ACS. E quatro agentes participaram da segunda etapa qualitativa da pesquisa, onde voluntariamente uma ACS de cada equipe se dispôs a participar. Essa segunda etapa teve a participação de apenas quatro, pois o estudo foi realizado em um momento de pandemia. As participantes foram recrutadas, a partir do convite para a participação na pesquisa.

As participantes foram informadas e esclarecidas verbalmente e por escrito sobre os objetivos do estudo e procedimentos da coleta de dados do trabalho. Aquelas que concordaram em participar assinaram o Termo de Consentimento Livre e Esclarecido.

Critérios de inclusão: atuar há mais de um ano na função de ACS na USF selecionada para o estudo, ter interesse e disponibilidade para participar da pesquisa. Foram excluídas as ACS que estavam de licença ou férias no período da coleta de dados.

A coleta dos dados ocorreu em três fases: Diagnóstico situacional; Ação; e Avaliação da ação. Inicialmente foi realizado o diagnóstico situacional, através de um formulário de entrevista do tipo estruturado para obter dados a respeito dos Conhecimentos, Atitudes e Práticas (CAP) das participantes da pesquisa referente aos marcos do desenvolvimento auditivo e de linguagem na primeira infância.

O instrumento de coleta de dados foi construído pelas pesquisadoras. Foram consideradas referências para cada uma das dimensões do método CAP, os seguintes aspectos:

Conhecimento - refere-se a relembrar fatos específicos (dentro do sistema educacional no qual o indivíduo está inserido) ou a habilidade para resolver os problemas de forma específica ou saber definir conceitos de acordo com a compreensão adquirida sobre uma determinada temática ou evento. Atitude - é ter opinião, predisposição, crença, sentimento, afetividade, relativamente incessantes, acerca de algo, de uma situação ou de pessoas. Está relacionado com o domínio afetivo, retrata a dimensão emocional. Prática - é a deliberação para realizar uma ação. Está relacionado aos domínios cognitivo e psicomotor, reforça uma dimensão social^(12:25)^.

O formulário foi constituído de dois blocos de variáveis. O primeiro, composto por variáveis sociodemográficas e profissionais e, o segundo bloco por variáveis referentes aos Conhecimentos, Atitudes e Prática.

Também foi realizado um grupo focal com as ACS participantes do estudo, conduzido no espaço da USF Vila União, antes e após a oficina.

O grupo focal é uma entrevista com um pequeno número de pessoas sobre um tópico específico que enfatiza a atenção por diferentes opiniões. Cada pessoa é estimulada a apresentar suas ideias e a ouvir e responder as outras, sendo assim o grupo focal tem como objetivo a sinergia entre as pessoas e não o consenso. A pesquisa do tipo grupo focal também proporciona centralização da discussão; informações em maior profundidade por conta da interação e influência do grupo; custos reduzidos; técnica e resultados facilmente entendidos; evidencia as diferenças de opiniões e contradições entre os participantes; possibilita resultados rápidos^(13)^.

O grupo foi conduzido por um moderador e um responsável pelo registro das falas. As respostas dadas pelo grupo foram gravadas e posteriormente transcritas.

Foram dirigidas às participantes perguntas orientadoras, organizadas a partir de quatro eixos de análise. Caso o grupo não respondesse satisfatoriamente, seriam dirigidas as perguntas auxiliares.

Para assegurar o anonimato, as entrevistadas foram identificadas pela sigla ACS e pelo número da entrevista. Para exemplificar: a primeira Agente Comunitária de Saúde entrevistada foi identificada como (ACS1), a segunda como (ACS2) e assim sucessivamente.

A ação da presente pesquisa foi desenvolvida por meio de uma oficina de apoio matricial, com estímulo à autonomia na construção do saber pelos ACS, com carga horária média de quatro horas. A oficina foi realizada a partir de uma explanação e discussão sobre os marcos de desenvolvimento da audição e linguagem e a conduta do ACS na visita domiciliar após a identificação desses marcos. As atividades foram programadas através de dinâmicas com intuito de descontrair e, ao mesmo tempo, envolver as integrantes com a proposta pedagógica, incentivando a participação e, de antemão, a reflexão do tema central da oficina (Quadro 1).

**Quadro 1:** Programa da oficina de apoio matricial sobre os marcos do desenvolvimento auditivo e de linguagem na primeira infância junto a Agentes Comunitários de Saúde

**Tema: “Marcos do desenvolvimento auditivo e de linguagem”**
Objetivos de aprendizagem:
Trabalhar os marcos de desenvolvimento da audição e linguagem.
Trabalhar a conduta na visita domiciliar após a identificação dos marcos de desenvolvimento.
Material:
a) Cartolina;
b) Etiquetas;
c) Canetas.
1º momento: Compartilhamento do conhecimento sobre os marcos do desenvolvimento auditivo e de linguagem na primeira infância
Inicialmente foi feita uma dinâmica através de colagem em uma cartolina. Foi apresentado as acs várias etiquetas de papel contendo os marcos auditivos e de linguagem. Em seguida elas tiveram que colar as etiquetas onde achava que era relativo aos marcos auditivos e no local que fosse relativo aos marcos de linguagem.
2º momento: Discussão sobre os marcos do desenvolvimento auditivo e de linguagem na primeira infância
A discussão foi realizada a partir do conhecimento prévio das ACS, onde através da cartolina que elas fizeram no primeiro momento iniciamos a nossa roda de conversa sobre cada marco do desenvolvimento.
3º momento: Momento de compartilhamento sobre a conduta na visita domiciliar após a identificação dos marcos de desenvolvimento
Esse momento foi realizado a partir de exemplos de situações problema vivenciadas no dia a dia pelas ACS durante a visita domiciliar onde a partir dos relatos tivemos a problematização de qual seria a conduta necessária.
4º momento: Dinâmica de fechamento
Cada participante definiu a oficina momento em uma palavra.

A coleta dos dados foi finalizada com a realização de um novo grupo focal, conduzido no espaço da USF Vila União, após a oficina, nos mesmos moldes do primeiro grupo focal, onde foram realizadas as mesmas perguntas orientadoras, acrescidas de perguntas referentes ao processo vivenciado na oficina.

Os dados qualitativos foram analisados através da análise de conteúdo, segundo Bardin^(14)^. A análise foi desenvolvida através das seguintes etapas: leitura flutuante das transcrições para compreender o todo e organizar os dados; identificação de categorias e subcategorias de análise nas falas dos sujeitos; descrição dos resultados; e interpretação dos resultados (questionamento da relação entre as categorias e subcategorias de análise e os objetivos propostos pelo estudo). Para a análise do questionário CAP se fez uso da estatística descritiva (distribuição de frequência absoluta e relativa). Onde todos os dados foram digitados e calculados através do programa Microsoft Excel, versão 2007.

## RESULTADOS

Participaram da pesquisa 18 ACS, todas do sexo feminino. A faixa etária predominante foi de 41 a 50 anos de idade (88,9%). Em relação à escolaridade, a maioria afirmou possuir ensino médio completo (72,2%). Cerca de 88,9% atua na função há nove anos ou mais (Tabela 1).

**Tabela 1:** Características sociodemográficas e profissionais dos agentes comunitários de saúde (n=18). Recife, Pernambuco, 2020

**Variáveis**	**N**	**%**
**Sexo**		
Masculino	0	0
Feminino	18	100%
**Faixa etária (em anos)**		
Até 40 anos	0	0
41 a 50 anos	16	88,9%
Mais de 60 anos	2	11,1%
**Escolaridade**		
Fundamental completo	1	5,6%
Ensino médio incompleto	1	5,6%
Ensino médio completo	13	72,2%
Superior incompleto	1	5,6%
Superior completo	2	11,1%
**Tempo que atua como ACS (em anos)**		
6 a 8 anos	2	11,1%
9 ou mais anos	16	88,9%
**TOTAL**	18	100%

A avaliação dos conhecimentos, atitudes e práticas das agentes comunitárias de saúde sobre os marcos do desenvolvimento auditivo e de linguagem na primeira infância demonstrou certa fragilidade de conhecimento sobre o tema, assim como uma prática sem embasamento (Tabela 2).

**Tabela 2:** Conhecimentos, Atitudes e Práticas dos Agentes Comunitários de Saúde sobre os marcos do desenvolvimento auditivo e de linguagem na primeira infância. Recife, Pernambuco, 2020

**VARIÁVEIS**	**RESPOSTA CAP**			
	**AFIRMATIVA**		**NEGATIVA**	
	**n**	**%**	**n**	**%**
**CONHECIMENTOS**				
Teste da orelhinha	17	94,12%	1	5,88%
Teste da linguinha	9	50%	9	50%
Hospitais que realizam a Triagem Auditiva Neonatal	18	100%	0	0%
Hospitais realizam o Teste da Linguinha	14	71,43%	4	28,57
Faz parte das atribuições do ACS as ações coletivas de saúde auditiva e de linguagem no território e na sala de espera.	15	83,3%	3	16,7%
Conhecimento que o desenvolvimento da fala da criança pode estar relacionado com a saúde auditiva que ela apresenta.	18	100%	0	0%
Conhecimento de que o desenvolvimento da fala da criança pode estar relacionado com a estimulação que ela recebe.	18	100%	0	0%
**ATITUDES**				
**Procurou obter formação sobre os marcos do desenvolvimento auditivo e de linguagem na primeira infância**	8	44,4%	10	55,6%
**Quem procurou, como obteve a formação**				
Fonoaudiólogo da equipe Nasf-AB	10	55,6%	8	44,4%
Folhetos, televisão, livros, Internet	8	44,4%	10	55,6%
**Sente-se capacitado para realizar orientações sobre desenvolvimento auditivo e de linguagem na primeira infância**	6	33,3%	12	66,7%
**Interesse em adquirir conhecimentos sobre o desenvolvimento auditivo e de linguagem na primeira infância**	18	100%	0	0%
**PRÁTICAS**				
Realiza ações de promoção à saúde auditiva e sobre o desenvolvimento da linguagem na primeira infância.	8	44,4%	10	55,6%
Utiliza os marcos do desenvolvimento auditivo e de linguagem da caderneta de saúde da criança.	4	22,2%	14	77,8%
Orienta sobre os fatores de risco para a saúde auditiva na primeira infância.	13	72,2%	5	27,8%
Orienta sobre os fatores de risco para o desenvolvimento da linguagem na primeira infância.	15	83,3%	3	16,7%
Questiona se os pais apresentam queixa quanto à audição ou a linguagem da criança durante as visitas domiciliares.	16	88,9%	2	11,1%
Identifica quando o usuário tem necessidades de assistência fonoaudiológica.	18	100%	0	0%
Ao suspeitar que a criança tem alguma dificuldade de comunicação, discute com sua equipe os sinais observados, a fim de tomarem um encaminhamento para o fonoaudiólogo do Nasf-AB	18	100%	0	0%
Sente necessidade de adquirir conhecimentos sobre os marcos do desenvolvimento auditivo e de linguagem.	18	100%	0	0%

Dentre as agentes comunitárias participantes, quatro participaram da segunda fase da pesquisa, de cunho qualitativo. Na análise de conteúdo sobre o conhecimento das ACS sobre a primeira infância, antes da participação na oficina de apoio matricial, foi identificada fragilidade nas falas sobre a temática (Quadro 2). Para elas, a primeira infância está ligada à idade da criança, citando apenas o recorte cronológico dessa etapa da infância, com diversidade nas faixas etárias apresentadas. Não foram mais abrangentes citando elementos característicos dessa fase.

**Quadro 2:** Categorias de análise antes e após oficina de apoio matricial, segundo núcleos de sentido referentes ao conhecimento dos agentes sobre os marcos do desenvolvimento auditivo e de linguagem na primeira infância

**Núcleos de sentido**	**Categorias de análise antes do Apoio Matricial**	**Categorias de análise após o Apoio Matricial**
Conhecimento sobre primeira infância	Tempo de vida da criança	Acompanhamento da criança dos 0 aos 5 anos
	0 a 5 anos de idade	Acompanhamento da criança desde a gestação até os 5 anos
	0 a 10 anos de idade	
Ações dirigidas à primeira infância na visita domiciliar	Orientação às famílias	Orientação às famílias
		Observar a fala e audição
		Ouvir os responsáveis, observar, orientar e fazer o encaminhamento.
Orientações para as famílias das crianças que estão na primeira infância	Cartão de vacina	Cartão de vacina
	Puericultura	Puericultura
	Amamentação	Amamentação
	Alimentação	Alimentação
	Marcos do desenvolvimento (engatinhar e balbuciar)	Marcos do desenvolvimento que constam na caderneta de vacina
Conhecimento sobre marcos do desenvolvimento auditivo	Inexistente	Identificar o som e localizar o som
Perguntas sobre o desenvolvimento da audição na visita domiciliar	Inexistência de perguntas na visita domiciliar	Perguntar a mãe se ele responde quando escuta um barulhozinho mais alto
	Assusta com barulho ou som alto?	
	Perguntas direcionadas à fala da criança	
Questionamentos das famílias sobre desenvolvimento da audição	Inexistência de perguntas por parte das famílias.	Não se aplica
	Relatam mais questões ligadas a linguagem (fala)	
Conhecimento sobre marcos do desenvolvimento da linguagem	Inexistente	A criança emite palavras com uma ou duas sílabas
		A criança emite uma frase com duas palavras ‘’mama água’’
	Língua presa	Repete palavras com duas sílabas (mama, papa)
		Emitir sons, emite sons e ri alto
Perguntas sobre o desenvolvimento da linguagem na visita domiciliar	Não realiza perguntas na visita	Sua criança já está emitindo sons, que tipo de som? Já consegue dizer mama, papa? Ta pedindo gaga?
	Pergunta de acordo com o tempo de vida da criança	Sua criança já fala , o que essa criança diz?
	Marcos do desenvolvimento (balbuciar, falar mama, papa)	Seu bebê tu já fizesses algum teste nele pra ver se ele ta escutando, se fala direitinho?
		Pergunto se ele emite algum som? Algum barulho? Já chama mamãe, já chama papai?
Questionamentos das famílias sobre desenvolvimento da linguagem	Tem famílias que questionam e outras que não	Não se aplica
	Quando os pais identificam algo, eles questionam	
Conduta do ACS frente ao não alcance dos marcos do desenvolvimento da criança	Repasse do caso para enfermeira ou médica	Orientar e trazer o caso para a equipe
	Repasse na reunião Nasf-AB +USF	Trazer o caso para a equipe de saúde e depois repassar para o Nasf-AB
	Agenda consulta com a médica	Trazer o caso para minha enfermeira ou médica
		Orientar, encaminhar e monitorar
Essa oficina de apoio matricial contribuiu para a sua prática profissional?	Não se aplica	Outra bagagem.
		Conhecimento a mais.
		Contribuição do aprendizado
		Segurança
		Enriquecimento de conhecimento

Após a participação na oficina percebeu-se que a idade continua ligada à primeira infância, porém as falas são mais assertivas para o acompanhamento da criança dos zeros aos cinco anos de idade e que esse acompanhamento pode acontecer desde a gestação.

O cartão de vacina foi identificado como referência principal para as ACS, porém as agentes trazem consigo um olhar fragmentado dos conhecimentos que são repassados no âmbito da saúde, majoritariamente.

Quando questionadas sobre as ações dirigidas na primeira infância às famílias de sua área de cobertura, há relato da realização de orientação para as famílias.

Após a participação na oficina essa fala ligada à realização de orientação é acrescida de afirmações direcionadas para a observação da fala e audição e realização de encaminhamentos.

Ouvir os responsáveis, observar, orientar e fazer o encaminhamento. Que no caso é o agendamento para os profissionais na unidade de saúde *(ACS 2).*

Já no que diz respeito ao tipo de orientação que seria dada, relatam o cartão de vacina, puericultura, amamentação e alimentação como sendo algumas das orientações. Após a oficina, as falas permanecem com o mesmo sentido com acréscimo dos marcos do desenvolvimento que estão disponíveis na caderneta de saúde da criança para que as ACS orientem as famílias.

ANTES:

É... primeiro lugar é orientar sobre a amamentação né? A importância da amamentação tudinho e orientar a vir frequentemente quando tiver marcada a puericultura para acompanhar o desenvolvimento da criança e orientar a mamadeira, chupeta essas coisas. E ver o cartão de vacina sempre pra ver se a vacina tá em dia *(ACS 1).*

APÓS:

É orientar né? Sempre tá orientando os pais pra que sempre veja a caderneta da vacina onde tem o desenvolvimento e crescimento da criança né? A gente tem que orientar eles e também encaminhar eles se a gente identificar algum, como é que se diz? Algum sinal né? Na criança, na fala, na audição *(ACS 1)*.

O conhecimento com relação aos marcos do desenvolvimento auditivo não apareceu nas respostas das agentes. Após a participação na oficina esses marcos aparecem na fala das agentes onde elas citam a identificação do som e a localização do som como sendo marcos auditivos.

ANTES:

eu num lembro não *(ACS 3).*

APÓS:

São localizar o som e identificar o som *(ACS 3).*

Percebe-se também na fala das agentes a inexistência de perguntas sobre a audição na visita domiciliar, onde as agentes relatam direcionar mais perguntas relacionadas à linguagem. Assim como também não há por parte das famílias perguntas direcionadas para questões de audição. Sempre há existência de perguntas relacionadas à linguagem.

Após a participação na oficina, as ACS direcionam em seus discursos perguntas para a visita domiciliar direcionadas à percepção da mãe sobre reações e respostas da criança quando escuta um barulho mais alto, ou se a criança se assusta ao escutar algum barulho.

ANTES:

Geralmente eu pergunto mais a gente pergunta mais da fala né? Ai as vezes é que assim a gente pergunta se a criança se assusta com barulho, som alto, mas geralmente mesmo a gente pergunta mais na fala e no andar da criança *(ACS 1).*

APÓS:

Perguntar à mãe, perguntar como é que tá né? É. Perguntar aos responsáveis se quando bate palma a criança de algum sinal de que está ouvindo, se quando cai alguma coisa se a criança tem alguma reação, se quando alguém tá falando como é que fica a expressão dela né, os olhos, o jeitinho da cabeça, perguntando aos responsáveis pra poder daí tirar algumas conclusões *(ACS 2).*

Já com relação aos marcos de desenvolvimento da linguagem as ACS trouxeram em seus relatos a inexistência de conhecimento perante os marcos. Mostrando mais uma vez, fragilidade de conhecimento sobre a temática. Após a participação na oficina elas trazem em suas falas alguns marcos de linguagem. Demonstrando assim que conseguiram abranger o conhecimento sobre os marcos de linguagem durante a oficina.

ANTES:

A linguagem papa, pepe, dada, querquer né? E a gente vai observando se vai evoluir ou se ele vai ficar só nisso ou então só apontar *(ACS 4) .*

APÓS:

São 8 marcos, tem como é que se diz? Emitir sons, emite sons e ri alto, é falar uma palavra, falar com duplicação de palavras, aos 5 anos ela já fala frases, ela já fala frases completas que você já entende *(ACS 3) .*

Relatam ainda que nem sempre costumam fazer perguntas sobre a linguagem durante suas visitas domiciliares e quando fazem direcionam as perguntas de acordo com a idade da criança. Já após a participação na oficina, trazem em suas falas perguntas direcionadas à emissão de sons e elaboração de palavras pela criança.

ANTES:

Eu pra ser sincera não. Nunca perguntei não *(ACS 1).*

APÓS:

Mãe o responsável né. Vou dar um nome! Maria a sua criança já está emitindo sons, que tipo de som? Já consegue dizer mama, papa? *(ACS 2).*

Já com relação aos questionamentos por parte dos familiares, elas relatam que muitos pais questionam, já outros não. As perguntas surgem mais quando os pais identificam algo em seus filhos.

Na minha área é muito relativo tem família que pergunta né? Ana oia ele já tá com tantos meses já fala bobagem. Outros nem assim, acha que é normal mesmo num falar, mas num é todas que pergunta não, são algumas. E eu acho que é as que tem um pouquinho mais de conhecimento, na verdade são essas que tentam buscar mais informações *(ACS 3).*

No que diz respeito à conduta das agentes perante aos casos que possam surgir no caso de uma criança não atingir algum marco do desenvolvimento, foi relatado o repasse do caso para enfermeira ou médica de sua equipe, relataram ainda que marcam consulta médica para que aquela criança seja avaliada pela médica e depois disso o caso seja então repassado para a equipe Nasf-AB. Percebe-se também, que as ACS não realizam nenhum trabalho educativo na unidade sobre a temática.

Após a oficina, as agentes relataram como conduta a realização no primeiro momento de orientações para as famílias, e encaminhamento do caso para sua equipe de saúde, se referindo à médica ou à enfermeira, e após isso repassar para a equipe Nasf-AB.

ANTES:

Passar pra enfermeira ou pra médica que elas entram em contato com o Nasf *(ACS 2).*

APÓS:

Eu trago o caso assim pra minha médica ou minha enfermeira né? Pra elas, pra depois quando tiver a reunião com o pessoal do Nasf a gente passar novamente o caso e a gente ficar acompanhando né? Ficar sempre indo na casa, vendo como é que tá o desenvolvimento e crescimento dessa criança né? *(ACS 1).*

Nas avaliações pré e pós-oficina, observaram-se diferenças quanto ao conhecimento das ACS referente aos marcos do desenvolvimento auditivo e de linguagem. Todas as ACS afirmam que a experiência contribuiu para o seu aprendizado, enriquecendo ainda mais a prática da visita domiciliar junto à população nos primeiros anos de vida. Um dos grandes aspectos de destaque foi o diferencial das categorias de análise sobre os marcos do desenvolvimento auditivo e de linguagem, no qual houve inexistência antes da oficina e relato após.

## DISCUSSÃO

A totalidade das ACS participantes do estudo foi do sexo feminino, possui mais de 41 anos de idade, ensino médio completo, e exerce a função há mais de nove anos. A predominância do sexo feminino foi verificada em resultados semelhantes obtidos através de alguns estudos, pelos quais, o sexo feminino era prevalente e a faixa etária era entre 40 anos ou mais^(15,16)^.

Quanto aos conhecimentos, atitudes e práticas das agentes comunitárias de saúde sobre os marcos do desenvolvimento auditivo e de linguagem na primeira infância, houve demonstração de certa fragilidade no conhecimento sobre o tema, assim como prática sem embasamento. Se faz necessário que o ACS tenha habilidades conceituais e atitudinais com relação à saúde da comunicação humana.

Nesse sentido, observa-se que há muito conhecimento por parte dos ACS, porém esta classe de trabalhadores necessita de capacitações inclusive da área fonoaudiológica^(17)^. Portanto, há necessidade de realizar capacitações para esses profissionais, a fim de que se tornem agentes potenciais no que diz respeito a educação, promoção e prevenção na saúde da comunicação humana.

Os achados revelam que o processo de apoio matricial junto aos ACS é fundamental para garantir uma atuação mais qualificada e engajada destes profissionais com os usuários do serviço e suas demandas. O compartilhamento de saberes, a prevenção, a promoção da saúde e o aprimoramento profissional de suas equipes são elementos que devem ser promovidos na APS, com o intuito de garantir um atendimento integral à população.

Corroborando com a perspectiva de que o apoio matricial é fundamental aos ACS, um estudo afirma que através do apoio matricial há mudanças na compreensão e prática dos profissionais, assim como mudanças organizacionais na USF e em sua relação com a rede de serviços, evidenciando assim que o apoio matricial é uma ferramenta de intervenção efetiva^(18)^.

Antes de participarem da oficina de apoio matricial, o conhecimento das ACS sobre a primeira infância trazia consigo uma perspectiva fragilizada dos conhecimentos repassados no âmbito da saúde, majoritariamente. Reitera-se que a primeira infância não é uma data, mas faz parte de todo um processo específico de desenvolvimento que o indivíduo passa num determinado período da vida.

Corroborando com esse aspecto, a PNAISC afirma que a primeira infância é o período de vida entre zero e cinco anos ou até completar os seis anos. No sentido de garantir que essa etapa seja vivida de forma saudável, preconiza-se o acompanhamento do desenvolvimento da criança com ações que perpassam todos os níveis de atenção: promoção, proteção, atendimento, detecção precoce e reabilitação de alterações que podem repercutir na sua vida futura^(19)^. Nesse contexto, então, se faz necessário, na prática cotidiana das ACS, o conhecimento sobre a PNAISC, uma vez que ela é orientadora das práticas de atenção à saúde da criança.

Desse modo, observam-se esses aspectos nas falas das ACS. Quando questionadas sobre a realização de acompanhamento das crianças e suas respectivas famílias através da visita domiciliar, bem como nas perguntas sobre a audição e linguagem, houve uma variação de respostas. Especificamente, percebeu-se a ausência de perguntas sobre audição e a maior frequência da existência de questionamentos sobre fala.

A respeito desse aspecto, há contribuição científica que aponta o relato dos ACS sobre a abordagem em suas visitas das temáticas: o desenvolvimento da fala, dificuldades de aprendizagem e dificuldades de atenção e concentração^(20)^. O estudo aponta ainda que os agentes têm a responsabilidade de, em suas visitas domiciliares, fazer o acompanhamento da saúde da criança e, para isso, devem utilizar como instrumento de base a Caderneta de saúde da criança, a qual possibilita o acompanhamento detalhado do desenvolvimento da audição e da linguagem.

Assim, é necessário repensar sobre as prioridades e importâncias que as equipes dão a esse tema, a frequência e as formas pelas quais esse assunto é posto em debate, os métodos de matriciamento junto aos ACS’s e atribuições que devem ser exercidas a partir desse diálogo nas equipes. Ressalta-se que os ACS são uma categoria singular pela sua inserção e pertença à comunidade, de modo a ter um contato direto com as famílias, e, nesse sentido, esperou-se um núcleo mais consistente de informações por parte da categoria.

Por outro lado, na tentativa de distanciamento de possíveis culpabilização sobre esses profissionais, ressalta-se o excesso de demandas a que esses trabalhadores são submetidos. Corroborando com essa perspectiva, um estudo evidenciou que a sobrecarga de trabalho é relatada pelos agentes como uma dificuldade para realizar visitas domiciliares, o que pode auxiliar na tentativa de buscar justificativas para a dificuldade desses profissionais exercerem de modo efetivo sua função nos quesitos analisados neste trabalho^(21)^. Além disso, sinaliza-se a falta de clareza das equipes quanto às principais atribuições dos ACS, deslocando-os para outras atividades que demandam tempo e dificultam o cumprimento das visitas domiciliares conforme o preconizado, ou até em maior frequência, em caso de necessidade.

Também nessa perspectiva, a prescrição das atribuições dos ACS precisa ser revista para uma melhor definição do papel desse trabalhador e dimensionamento de suas ações, de acordo com os recursos disponibilizados, evitando, sobretudo, o desvio de funções^(22)^. Apesar das fragilidades - sobrecarga de trabalho, desvio de funções e falta de capacitação - as agentes, ao terem contato com as crianças e perceberem uma possível intervenção, desenvolvem orientações. No entanto, a prevenção a partir de informações passadas previamente às mães das crianças é escassa.

Outra questão identificada foi a forma como os ACS repassam os casos para sua equipe de saúde e, em seguida, para a equipe Nasf-AB. Demonstra-se, dessa maneira, uma fragilidade na organização do trabalho entre os agentes e as equipes da ESF, haja vista o não relato da discussão do caso em equipe. Um estudo indica que a organização do trabalho das equipes da ESF deve ser revista de forma a permitir que o ACS tenha um espaço de diálogo fortalecido com os demais integrantes da equipe^(22)^. De fato, quando o trabalho em equipe ocorre de forma mais horizontalizada, com integração entre seus membros, isso se reflete, positivamente, no trabalho do ACS.

Identificou-se como limitação do presente estudo a diferença no quantitativo de ACS que participou do questionário CAP e dos grupos focais. Constatou-se também escassez de literatura científica nessa temática sobre o conhecimento dos agentes comunitários de saúde no processo de apoio matricial sobre a identificação dos marcos do desenvolvimento auditivo e de linguagem na primeira infância no contexto da visita domiciliar.

A continuidade de pesquisas abordando essa temática é essencial para o acompanhamento do desenvolvimento auditivo e de linguagem na primeira infância. Desta forma, sugere-se que novos estudos sejam realizados, com o objetivo de capacitar cada vez mais agentes comunitários de saúde no que diz respeito aos marcos de desenvolvimento.

## CONCLUSÃO

Os resultados obtidos com este estudo permitem inferir a fragilidade no conhecimento sobre os marcos do desenvolvimento auditivo e de linguagem na primeira infância por parte das ACS. Já no pós-oficina o conhecimento foi ampliado. Uma questão a ser destacada é o fato de que as agentes não se sentiam capacitadas para a realização de orientações sobre os marcos auditivos e de linguagem. Contudo, foi apontada a necessidade de atualizar os profissionais sobre o tema, afirmando assim a importância do apoio matricial para esses profissionais.

O debate sobre o conhecimento dos ACS a respeito da identificação dos marcos de desenvolvimento auditivo e de linguagem na primeira infância é necessário, devido ao fato de que esses profissionais possuem um contato privilegiado com a população, conseguindo perceber os possíveis entraves que permeiam o desenvolvimento da audição e da fala apresentados pelas crianças nos anos iniciais de sua trajetória de vida.
